# Associations between weight stigma and exercise avoidance motivation among college students: exploring the roles of internalized weight stigma and social anxiety

**DOI:** 10.3389/fpsyg.2025.1655699

**Published:** 2025-10-20

**Authors:** Qingqing Li, Ling Shao, Hansen Li, Yuping Zhu, Yun Li

**Affiliations:** ^1^College of Physical Education, Southwest University, Chongqing, China; ^2^College of Physical Education, Chongqing College of Humanities, Science and Technology, Chongqing, China; ^3^College of Physical Education, Sichuan Agricultural University, Ya'an, Sichuan, China

**Keywords:** weight stigma, exercise avoidance, internalized weight stigma, social anxiety, exercise behavior

## Abstract

**Background:**

Weight stigma is linked to a range of adverse outcomes, including reduced engagement in physical activity, yet the psychological pathways underlying these associations remain insufficiently understood. This study examined the relationship between weight stigma and motivation to avoid exercise, with a particular focus on the mediating roles of internalized weight stigma and social anxiety.

**Methods:**

A total of 1,397 Chinese university students (aged 17–25) were recruited via the online survey platform Sojump. Participants completed validated questionnaires assessing weight stigma, exercise avoidance motivation, internalized weight stigma, and social anxiety. Structural equation modeling was employed to evaluate the hypothesized relationships. The study protocol was approved by the Ethics Committee of the College of Physical Education, Southwest University.

**Results:**

Correlational analyses suggested a partial mediation model, in which weight stigma contributed to exercise avoidance motivation both directly and indirectly via internalized weight stigma and social anxiety. Specifically, higher weight stigma was linked to greater internalized weight stigma (*β* = 0.45, *p* < 0.001), which, in turn, was associated with increased social anxiety (*β* = 0.40, *p* < 0.001), ultimately leading to heightened motivation to avoid exercise (*β* = 0.33, *p* < 0.001).

**Limitations:**

The limitations of this study include the use of a cross-sectional design, which precludes causal inferences, and the relatively small number of individuals with a high BMI in the sample, which may limit the broader applicability and generalizability of the findings.

**Conclusion:**

We recommend that interventions focus on reducing internalized weight stigma and social anxiety to help individuals overcome exercise avoidance motivation, thereby promoting physical activity and improving mental health.

## 1 Introduction

Weight stigma, defined as “society’s devaluation and vilification of individuals with excess weight” (Tomiyama, 2014), has been consistently linked to psychological distress and maladaptive health behaviors. Exercise avoidance motivation denotes an individual’s tendency to actively avoid exercise (More et al., 2019). Research has demonstrated that experiences of weight stigma are associated with diminished exercise participation and lower motivation for physical activity, particularly among individuals with higher body weight (Schvey et al., 2017; Vartanian, 2015; Vartanian and Novak, 2011; Vartanian and Shaprow, 2008). A systematic review found that both weight stigma and internalized weight stigma are closely associated with a decrease in physical activity levels. This relationship is often not a direct effect but rather an indirect influence mediated through a series of psychological and behavioral mechanisms that impact individuals’ participation in physical activity (Pearl et al., 2021). Furthermore, the research showed that internalized weight stigma partially mediated the relationship between weight stigma and exercise behavior (Bidstrup et al., 2022). Similarly, individuals with pronounced internalized weight stigma not only exhibit an intensified fear of negative evaluation by others but also endorse weight-related stereotypes, such as the belief that ‘higher-weight individuals have less willpower and are less deserving of a fulfilling social life.’ This self-stigmatization fosters feelings of diminished self-worth, increased self-consciousness, and perceived incompetence, which in turn may contribute to greater vulnerability to depression, anxiety, body dissatisfaction, and disordered eating behaviors (Emmer et al., 2020; Hilbert et al., 2014; Schvey et al., 2017). These psychological burdens can further reinforce avoidance of physical activity, creating a cycle of distress and inactivity. While prior studies have established a link between weight stigma and exercise avoidance motivation (Ajibewa et al., 2024; Bevan et al., 2021; Han et al., 2018), the precise psychological mechanisms underlying this relationship remain insufficiently understood. Given the profound implications for mental health and physical well-being, further research is warranted to elucidate the internalization process of weight stigma and its specific pathways influencing exercise avoidance motivation. This is particularly relevant for college students, among whom experiences of weight stigma may discourage participation in sports and physical activity (Ajibewa et al., 2024). Understanding these mechanisms has important implications for developing interventions and strategies to promote student engagement in physical activity.

The association between weight stigma and exercise engagement is profoundly shaped by internalized weight stigma, which has been identified as a critical psychological mediator (Bevan et al., 2021; Pearl et al., 2015). Internalized weight stigma occurs when individuals assimilate external weight-related biases, transforming societal criticism into self-directed shame and self-judgment (Palmeira et al., 2018). This internalized distress not only diminishes self-efficacy and influences behavioral decisions but also deepens psychological suffering (Pearl et al., 2023). As individuals internalize weight stigma, they become increasingly preoccupied with perceived external scrutiny of their body size, fostering negative self-appraisals that ultimately impair their motivation and willingness to engage in physical activity. Beyond internalized stigma, social anxiety may serve as another pivotal psychological mechanism underpinning the relationship between weight stigma and exercise avoidance (Vartanian and Novak, 2011). Research indicates that individuals with higher body weight are more likely to experience negative judgments related to their appearance and body size, which can lead to stronger anxiety in social situations (Gilbert and Miles, 2014). A hallmark of social anxiety is the tendency toward avoidance behaviors (Avramchuk et al., 2022; Heeren and McNally, 2018; Rinck et al., 2010; Wong and Moulds, 2011), which manifests as withdrawal from situations where individuals fear scrutiny or criticism (Morrison and Heimberg, 2013). In exercise-related environments—where bodily appearance is often visible and subject to evaluation—this fear becomes particularly salient, reinforcing avoidance of physical activity and further undermining exercise motivation. Moreover, the interplay between internalized weight stigma and social anxiety may establish a self-reinforcing cycle (Ratcliffe and Ellison, 2015). Experiencing weight stigma elicits intense negative emotional responses (Tomiyama, 2014), which, in turn, heighten social anxiety. This anxiety amplifies avoidance tendencies, further entrenching disengagement from physical activity. As a result, internalized weight stigma and social anxiety may function as interconnected mediators in the relationship between weight stigma and exercise avoidance, perpetuating psychological distress and physical inactivity.

**Figure 1:**
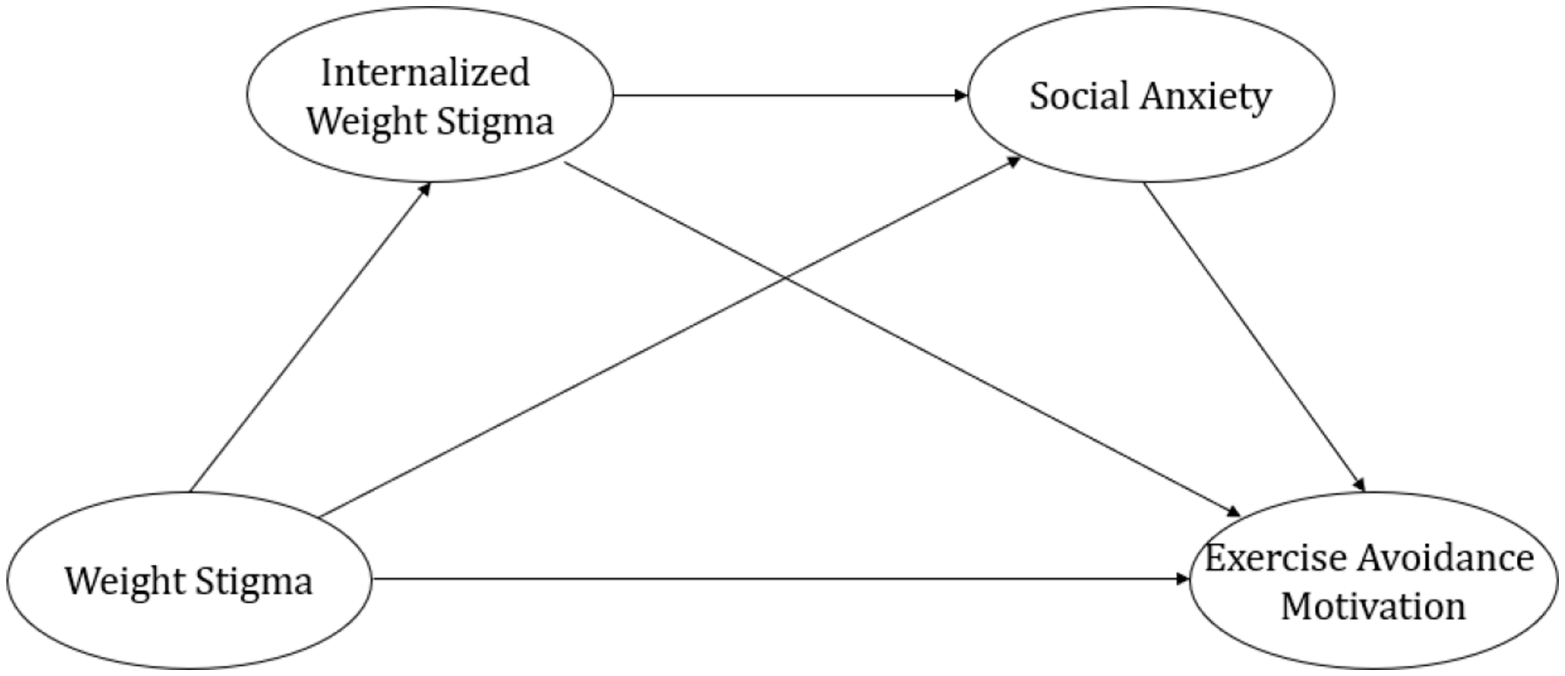
The conceptual model.

This study seeks to elucidate the psychological mechanisms underlying the association between weight stigma and exercise avoidance motivation, with a particular focus on the mediating roles of internalized weight stigma and social anxiety. Grounded in existing empirical findings and theoretical frameworks, we propose a conceptual model (Figure 1) that delineates the pathways through which weight stigma influences exercise avoidance motivation. Specifically, we hypothesize the following: (a) weight stigma is positively associated with exercise avoidance motivation among university students; (b) internalized weight stigma mediates the relationship between weight stigma and exercise avoidance motivation; (c) social anxiety serves as an additional mediator in this relationship; and (d) internalized weight stigma and social anxiety operate sequentially, forming a dual mediation pathway through which weight stigma contributes to exercise avoidance motivation.

## 2 Method

### 2.1 Participants

A total of 1,411 university students were recruited via the Chinese survey platform Sojump through campus announcements, class group messages, and social media posts to ensure a broad sample. The study focused on students aged 17–25 years, the typical age span of university students. After providing informed consent, participants completed a questionnaire on demographics (age, gender, height, weight, grade, monthly living expenses) and psychological variables (weight stigma, internalized weight stigma, social anxiety, exercise avoidance motivation). Invalid responses were excluded, yielding 1,397 valid cases (67.2% female), with a mean age of 19.61 years (SD = 1.26, range = 17–25) and mean BMI of 20.84 kg/m^2^ (SD = 3.29, range = 12.47–38.05). Based on World Health Organization (WHO) criteria, 23.5% were underweight (BMI < 18.5), 66.9% normal (18.5–24.9), 8.0% overweight (25.0–29.9), and 1.6% obese (≥30.0). As detailed in Table 1, the study was approved by the Ethics Committee of the College of Physical Education, Southwest University (TY20240306007).

**Table 1:** Demographic characteristics and descriptive statistics of the total sample (*n* = 1,397).

Variables	*n* (%)	Mean (SD)	Range	Skewness	Kurtosis
Gender					
Men	458 (32.8)				
Women	939 (67.2)				
Grade					
Freshman	639 (45.7)				
Sophomore	579 (41.4)				
Junior	73 (5.2)				
Senior	106 (7.6)				
Monthly living expenses					
<1,000¥	110 (7.9)				
1,000–2,000¥	1,090 (78.0)				
2,000–3,000¥	157 (11.2)				
>3,000¥	40 (2.9)				
BMI (kg/m^2^)		20.84 (3.29)	12.47–38.05	1.30	2.86
Underweight (<18.5)	328 (23.5)				
Normal (18.5–24.9)	934 (66.9)				
Overweight (25.0–29.9)	112 (8.0)				
Obese (≥30.0)	23 (1.6)				
Age (years)		19.61 (1.26)	17–25	0.97	1.37
Weight stigma (SSI-B)		14.54 (9.57)	10–80	3.52	14.06
Internalized weight stigma (WBIS-M)		29.76 (12.29)	11–73	0.67	−0.25
Social anxiety (SPIN)		45.54 (16.91)	17–85	−0.11	−0.62
Exercise avoidance motivation (EAMS)		20.98 (10.15)	8–56	0.64	−0.03

BMI, body mass index; SSI-B, Stigmatizing Situations Inventory-Brief; WBIS-M, Modified Weight Bias Internalization Scale; SPIN, Social Phobia Inventory; EAMS, Exercise Avoidance Motivation Scale.

### 2.2 Measures

#### 2.2.1 Weight stigma

Weight stigma experiences were measured with the Chinese version of Vartanian’s (2015) Stigmatizing Situations Inventory—Brief (SSI-B). This 10-item measure evaluates the frequency of weight-based stigma encounters across medical, occupational, and interpersonal contexts (e.g., not being hired due to one’s weight, shape, or size; overhearing derogatory remarks in public). Responses were originally rated on a 10-point scale (1 = never to 10 = every day); however, for this study, the scale was modified to an 8-point format by excluding the response options “a few times a month” and “every day.” The measure demonstrated excellent internal consistency, with a Cronbach’s alpha of 0.93.

#### 2.2.2 Internalized weight stigma

Pearl and Puhl (2014) developed the Weight Bias Internalization Scale (WBIS-M), an 11-item measure designed to assess the extent to which individuals endorse and internalize negative weight-related attitudes as self-relevant and valid. In this study, we utilized the Chinese version of the WBIS-M, with participants rating each item on a 7-point Likert scale (1 = strongly disagree to 7 = strongly agree). Higher scores reflect greater internalization of weight bias. The scale demonstrated good internal consistency in the present sample (Cronbach’s *α* = 0.87).

### 2.3 Social anxiety

The Social Phobia Inventory (SPIN) is a 17-item measure designed to assess social anxiety, with its Chinese version used in this study (Connor et al., 2000; Tsai et al., 2009). Participants rated each item on a 5-point Likert scale (1 = not at all to 5 = extremely), with higher scores reflecting greater levels of social anxiety. The SPIN evaluates fear, avoidance, and physiological discomfort in various social contexts. In the present study, the scale demonstrated excellent internal consistency (Cronbach’s *α* = 0.96).

### 2.4 Exercise avoidance motivation

Exercise avoidance motivation refers to an individual’s reluctance or negative attitude toward engaging in physical exercise. This construct was assessed using the Chinese version of the Exercise Avoidance Motivation Scale (EAMS; Vartanian and Novak, 2011), which comprises eight items. Example items include: “I avoid going to public places because I fear people will comment on my body,” and “I avoid looking in the mirror to prevent focusing on my weight.” Participants rated their agreement with these statements on a 7-point Likert scale, ranging from 1 (strongly disagree) to 7 (strongly agree). The final score was calculated as the mean of all item scores, with higher scores indicating a stronger motivation to avoid exercise. In the current sample, the scale demonstrated strong internal consistency, with a Cronbach’s alpha of 0.88.

### 2.5 Data analysis

Descriptive statistics, correlations, and mediation analyses were conducted using SPSS 25. Means and standard deviations were calculated for continuous variables, and frequencies for categorical variables. Pearson correlations examined associations among weight stigma, internalized weight stigma, social anxiety, and exercise avoidance motivation. A chained mediation analysis was conducted using the PROCESS macro (Hayes et al., 2017) to test whether internalized weight stigma and social anxiety sequentially mediated the relationship between weight stigma (X) and exercise avoidance motivation (Y). Following prior research indicating that gender is related to both weight stigma and exercise behaviors and may confound these associations (Sattler et al., 2018), the present study included gender, age, BMI, and monthly living expenses as control variables. Indirect effects were evaluated with 5,000 bootstrap resamples and 95% bias-corrected confidence intervals; significance was determined when the interval did not include zero.

## 3 Result

### 3.1 Correlation analysis

As presented in Table 2, all four variables were positively correlated with one another (all *p*-values < 0.01). Specifically, higher weight stigma among university students was associated with greater internalized weight stigma (*r* = 0.46), increased social anxiety (*r* = 0.26), and heightened motivation to avoid physical activity (*r* = 0.41). Moreover, stronger internalized weight stigma was linked to higher levels of social anxiety (*r* = 0.43) and a greater tendency to avoid physical activity (*r* = 0.61). In addition, elevated social anxiety was found to be positively correlated with stronger motivation to avoid physical activity (*r* = 0.54).

**Table 2:** Correlations between study variables (*n* = 1,397).

Variables	1	2	3	4	5	6
Weight stigma	–					
Internalized weight stigma	0.46**	–				
Social anxiety	0.26**	0.43**	–			
Exercise avoidance motivation	0.41**	0.61**	0.54**	–		
Gender	0.01	−0.01	−0.04	−0.05	–	
Age	0.03	−0.01	−0.02	−0.02	−0.01	–
BMI	0.05	0.12**	−0.01	0.06*	0.24**	−0.01

BMI, body mass index; *p < 0.05, **p < 0.01. Gender: 0 = men, 1 = women.

### 3.2 Structural model

**Figure 2:**
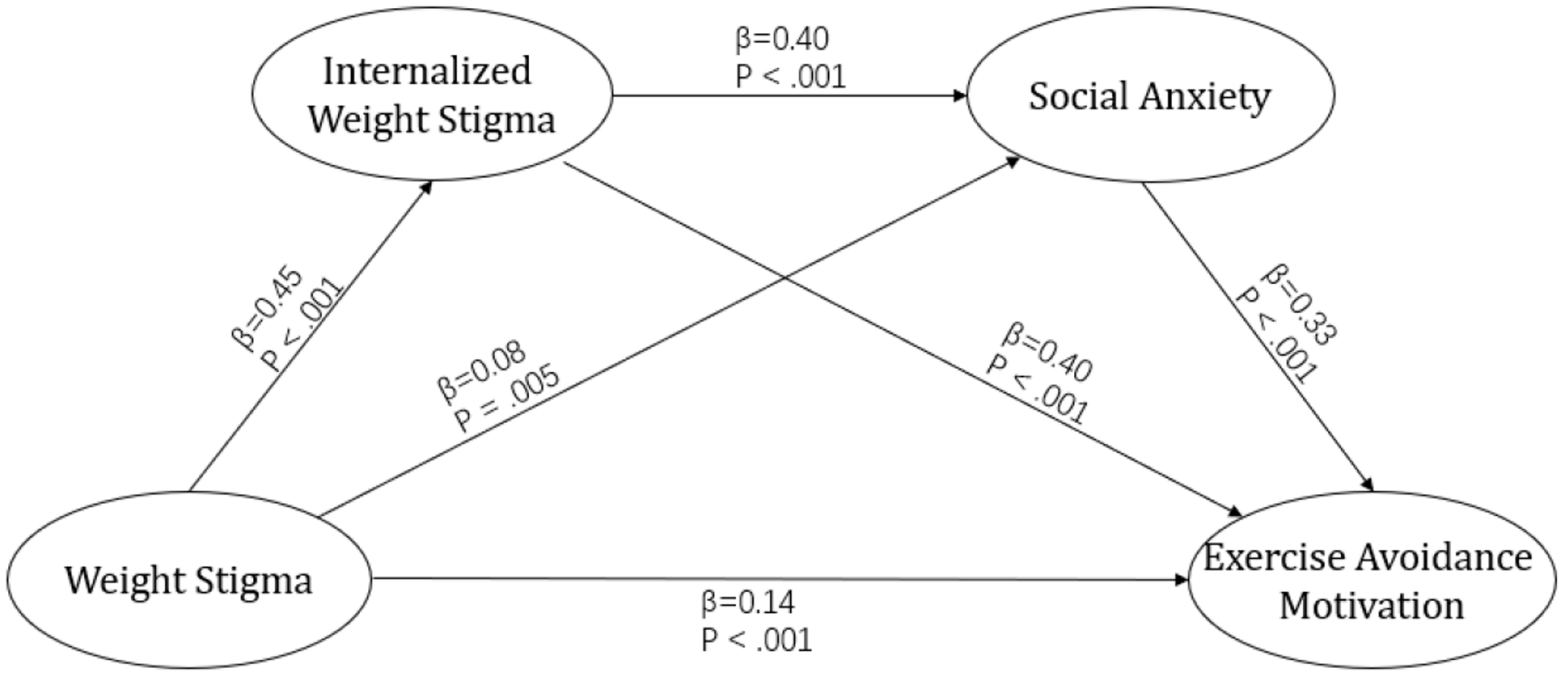
The statistical model, incorporating gender, age, BMI, and monthly living as covariates, included standardized regression weights for analysis.

As illustrated in Figure 2, the structural model analysis, after accounting for covariates, revealed a significant association between weight stigma and internalized weight stigma, social anxiety, and avoidance of physical activity among college students. Furthermore, the direct effect of weight stigma on physical activity avoidance was significant, indicating that the indirect effects of internalized weight stigma and social anxiety served as partial mediators in this relationship.

As presented in Table 3, the mediation effect was evaluated using the bootstrap method. The findings revealed that the indirect effect through internalized weight stigma was 0.18 (95% CI: 0.16–0.21), highlighting the significant mediating role of internalized weight stigma in the relationship between weight stigma and exercise avoidance (weight stigma → internalized weight stigma → exercise avoidance). Furthermore, the indirect effect through social anxiety was 0.03 (95% CI: 0.01–0.04), indicating a notable mediating effect of social anxiety (weight stigma → social anxiety → exercise avoidance). Additionally, the sequential pathway involving both internalized weight stigma and social anxiety produced an indirect effect of 0.06 (95% CI: 0.05–0.07), signifying a robust serial mediation effect (weight stigma → internalized weight stigma → social anxiety → exercise avoidance).

**Table 3:** Estimates of standardized indirect effects.

Indirect effects	Point estimate	95% CI	SE
Path 1: WS → IWS → EAM	0.18	0.16–0.21	0.01
Path 2: WS → SA → EAM	0.03	0.01–0.04	0.01
Path 3: WS → IWS → SA → EAM	0.06	0.05–0.07	0.01

CI, confidence interval; SE, standard error; WS, weight stigma; IWS, internalized weight stigma; EAM, Exercise avoidance motivation; SA, social anxiety.

### 3.3 Sensitivity analyses

To assess the robustness of our findings, we conducted a sensitivity analysis by reordering the sequence of internalized weight stigma and social anxiety (i.e., weight stigma → social anxiety → internalized weight stigma → exercise avoidance). This alternative model was tested to determine whether the independent and sequential indirect effects continued to hold significance. Supplementary Figure 1 illustrates the statistical model with standardized regression weights after reordering, while Supplementary Table 1 presents the unstandardized estimates of the indirect effects. The results confirmed that the indirect effects of internalized weight stigma and social anxiety still exist, underscoring the stability of these psychological pathways.

## 4 Discussion

This study sought to explore the mediating roles of internalized weight stigma and social anxiety in the relationship between weight stigma and physical activity avoidance motivation among a large sample of Chinese university students. The results aligned with the proposed hypotheses, revealing that weight stigma not only directly impacted students’ motivation to avoid physical activity but also exerted indirect effects through internalized weight stigma and social anxiety. Specifically, higher levels of weight stigma were associated with increased internalized weight stigma, which, in turn, heightened social anxiety, ultimately strengthening the motivation to avoid physical activity.

Specifically, weight stigma was found to be positively correlated with exercise avoidance motivation, indicating that higher levels of weight stigma may diminish motivation to engage in physical activity (Horenstein et al., 2021; Vartanian and Shaprow, 2008). Moreover, the association between internalized weight stigma and motivation to avoid exercise appeared more direct than the link between weight stigma and exercise avoidance motivation (Bevan et al., 2021; Pearl and Puhl, 2016), with internalized weight stigma demonstrating a more robust association with exercise avoidance. This aligns with previous research indicating that internalized weight stigma is a more reliable predictor of adverse health outcomes than objective experiences of weight stigma alone (Latner et al., 2014). Such findings suggest that experiences of weight discrimination may lead individuals to internalize negative beliefs about their weight, which in turn diminishes enjoyment of physical activity and fosters avoidance of exercise and physical activity (Smith et al., 2024). Notably, research has shown that women experience weight stigma more frequently than men. For women, heightened weight stigma is often associated with decreased autonomous motivation for physical activity and an overall decline in physical activity levels, with these effects typically mediated through indirect pathways. In contrast, men who frequently experience weight stigma may display higher levels of walking and more frequent participation in vigorous physical activity, with a direct influence on their behavior (Sattler et al., 2018). Moreover, in various social domains, such as employment, education, and romantic relationships, the impact of weight bias is far more pronounced for women than for men. Overweight or obese women are more likely to encounter discriminatory treatment than their lean counterparts or men, irrespective of their body size (Fikkan and Rothblum, 2012). This discrepancy may be attributed to the more rigid beauty and body standards imposed on women, as compared to men (Fikkan and Rothblum, 2012; Saguy, 2012), making women more susceptible to internalizing weight stigma. Additionally, our study’s findings align with previous research showing that social anxiety is associated with experiences of weight stigma and with exercise avoidance (Horenstein et al., 2021).

Notably, the indirect effects of internalized weight stigma and social anxiety were found to be partial, suggesting the involvement of additional psychological mechanisms. One such factor may be body image concerns, particularly the fear of negative evaluation related to one’s appearance (Bevan et al., 2021). Heightened appearance-related concerns could further amplify anxiety about being judged or criticized in exercise settings, reinforcing avoidance behaviors toward physical activity. Sensitivity analyses further demonstrated that reversing the order of internalized weight stigma and social anxiety did not alter the significance of the three indirect pathways, underscoring the robustness of these mediating effects. This finding reinforces the stability of the indirect role played by internalized weight stigma and social anxiety in the association between weight stigma and exercise avoidance motivation. Additionally, our results revealed that while BMI was not directly associated with weight stigma, it was significantly and positively correlated with internalized weight stigma. Individuals with higher BMI may be more susceptible to internalizing societal weight-related stereotypes, leading to heightened self-criticism and feelings of inferiority (Carter et al., 2022). Although weight stigma itself may not fluctuate with BMI, individuals with higher BMI often experience greater societal pressure and a heightened risk of stigmatization, increasing their vulnerability to internalized negative body perceptions (Farhat, 2015).

A growing body of research indicates that weight stigma originates primarily from external negative evaluations or discrimination based on body size, whereas internalized weight stigma emerges when individuals adopt these external judgments as self-perceptions (Durso and Latner, 2008; Pearl and Puhl, 2016; Ratcliffe and Ellison, 2015). Our findings suggest that when individuals internalize negative verbal comments or experiences of weight-based teasing, the detrimental effects of weight stigma are exacerbated. This underscores the potential existence of an “intervention window,” during which targeted interventions may help attenuate the adverse psychological and behavioral consequences of weight stigma. For instance, Acceptance and Commitment Therapy (ACT) has shown promise in reducing internalized weight stigma, though further empirical validation of its efficacy remains necessary (Griffiths et al., 2018). Future research should explore why some individuals are more susceptible to internalizing weight stigma than others, identify populations at heightened risk, and further investigate the psychological mechanisms that contribute to its negative impact on physical activity engagement (Pearl et al., 2015). Moreover, the severity of weight stigma may be closely linked to factors such as psychological resilience, social support, and self-efficacy (Meadows and Bombak, 2019). Individuals who experience heightened weight stigma often perceive themselves as having lower physical competence, and exposure to weight-based discrimination can undermine the development of exercise self-efficacy (Meadows and Bombak, 2019). Thus, interventions should not only focus on reducing weight stigma itself but also emphasize fostering psychological resilience and enhancing self-efficacy to promote long-term engagement in physical activity.

This study has several limitations. First, its cross-sectional design prevents conclusions about directionality or causality among weight stigma, internalized stigma, social anxiety, and exercise avoidance. Longitudinal and experimental research is needed to clarify these relationships. Second, only 9.5% of participants had a BMI above 25 and 1.6% exceeded 30, limiting representation of higher-BMI individuals. However, most participants were normal or underweight yet still reported notable levels of weight stigma, suggesting that stigma also reflects cultural pressures toward thinness. Such pressures may lead even those with objectively low BMI to internalize negative beliefs and experience social anxiety, reducing motivation for physical activity. Future research should examine these dynamics in larger and more diverse samples, including both overweight and underweight groups. Third, reliance on self-report may introduce recall or social desirability bias, particularly for sensitive constructs such as weight stigma and exercise. Objective measures, such as wearable activity trackers, could improve accuracy. Finally, gender differences should be considered. Prior work shows that men sometimes increase activity in response to stigma, whereas women often decrease it (Sattler et al., 2018). Although gender was controlled for, future studies could test gender as a moderator or investigate gender-specific pathways.

## 5 Conclusion

In conclusion, drawing from a large sample of Chinese university students, this study sheds light on the psychological mechanisms linking weight stigma to exercise avoidance motivation, emphasizing the mediating roles of internalized weight stigma and social anxiety. By extending our understanding of weight stigma beyond its direct effects, these findings underscore the critical influence of psychological factors in shaping exercise behavior. Moreover, the results highlight the importance of targeted interventions aimed at mitigating internalized weight stigma and social anxiety to reduce exercise avoidance and promote physical activity engagement.

## Data Availability

The original contributions presented in the study are included in the article/Supplementary material, further inquiries can be directed to the corresponding author/s.
