# Supplementary material for: Associations between weight stigma and exercise avoidance motivation among college students: exploring the roles of internalized weight stigma and social anxiety
Source: Front Psychol. 2025 Oct 20;16:1655699. doi: 10.3389/fpsyg.2025.1655699 (PMC12581209; doi:10.3389/fpsyg.2025.1655699)
Supplement: Supplementary file 1 [file Supplementary_file_1.docx]

Supplementary Material


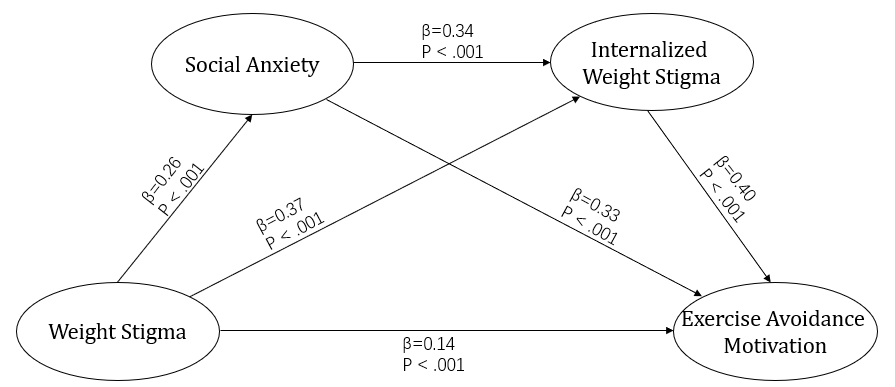


**Supplementary Figure 1.** A statistical model with standardized regression weights was used with gender, age, BMI, and monthly living as covariates.

| Supplementary Table 1. Estimation of unstandardized indirect effects. | | | |
| --- | --- | --- | --- |
| Indirect effects | Point estimate | 95% CI | SE |
| Path 1: WS → SA → EAM | .09 | .07–.10 | .01 |
| Path 2: WS → IWS → EAM | .15 | .13–.17 | .01 |
| Path 3: WS → SA → IWS → EAM | .04 | .03–.04 | .01 |
| Notes. CI = confidence interval, SE = standard error, WS = weight stigma, SA = social anxiety, EAM = Exercise avoidance motivation, IWS = internalized weight stigma . | | | |
